# A qualitative study of daily activities that inform a process-based model of well-being among Singaporean adults

**DOI:** 10.1186/s41687-024-00690-3

**Published:** 2024-01-31

**Authors:** Jumana Hashim, Huso Yi, Pin Sym Foong, E Shyong Tai, Robert P Nolan

**Affiliations:** 1 Saw Swee Hock School of Public Health, National University of Singapore, National University Health System, 12 Science Drive 2, #09-01W, Singapore, 117549 Singapore; 2 Division of Endocrinology, University Medicine Cluster, National University Hospital, Singapore, Singapore; 3 Behavioural Cardiology Research Unit, University Health Network, Toronto, ON Canada

**Keywords:** Well-being, Life aspirations, Qualitative study

## Abstract

**Background:**

Individual appraisals of personal well-being consider a spectrum of constructs including the reflections on the degree to which one’s life is ultimately secure, meaningful, or valued in the context of dynamic changes in their bio-psycho-social environments. Standardized questionnaires for well-being evaluate an ideal state of health which is operationally defined by abstract constructs. Patient reports describe well-being as a more dynamic construct that relates to how they adapt to successive changes in their life situations. While response shift theory addresses this dynamic nature, little is known about how personal well-being is pursued as personal aspirations evolve. In this study, we identify regularly practiced goal-directed activities reported to contribute to the pursuit of personal well-being. We then propose a taxonomy of these reported activities to inform a process-based model for well-being.

**Methods:**

Purposive sampling was conducted with individuals, 30 to 60 years of age, with diverse ethnicities reflecting the Singaporean population. Individual semi-structured interviews were administered with the primary question: “What are the things or activities you have done in the last two weeks that made you feel like you had a good day?” Probes explored the personally salient meaning of each activity. A thematic approach was used for data analysis followed by a framework analysis to categorize the activities into major themes.

**Results:**

We interviewed 40 individuals comprised of 60% female participants. Thematic analysis identified eight types of goal-directed activities, which were categorized under three major themes: (i) Self-Affirming Care through individual self-care and maintaining interpersonal relationships, (ii) Achievement-Focused Tasks as indicated by work-related accomplishments and developing a skill or hobby, (iii) Affiliative Growth and Support as reflected through religious practices and community services. Planned physical activity contributed to self-affirming care and achievement-focused tasks. Social affirming roles were associated with both achievement-focused tasks and affiliative growth and support.

**Conclusions:**

In the dimensions of goal-directed activities identified, there is close alignment between Self-Affirming Care and attachment theory; Achievement-Focused Tasks and self-determination theory; and Affiliative Growth and Support and social mattering. These findings can contribute to a comprehensive process-based model of well-being which is more closely aligned to patient-report appraisals of personal well-being.

**Supplementary Information:**

The online version contains supplementary material available at 10.1186/s41687-024-00690-3.

## Introduction

A casual inquiry in daily interactions about whether someone is well, or doing well, refers to a construct of personal well-being that is radically open-ended. The response to this question can include one’s appraisal of the quality of their cognitive-emotional experience, physical symptoms, environmental conditions, social interactions and relationships, degree of satisfaction with life, as well as their ability to progress towards goals that promote personal growth and flourishing [1–3]. The appraisal of personal well-being can also extend to existential reflections on the degree to which one’s life is ultimately secure, meaningful, or valued in the context of dynamic changes in their bio-psycho-social environments [4].

Contemporary psychometric assessments commonly evaluate well-being by comparing an individual’s profile to an idealized state in which there is an absence of pain, emotional distress, or physical limitations. These psychometric questionnaires incorporate at least three core philosophical themes in the operational definition of well-being: (i) hedonic features associate well-being with positive vs. negative mood, affect, and physical symptoms, (ii) eudaimonic well-being interprets well-being as individual flourishing where one is able to actualize their skills and personal competencies, and (iii) desire-satisfaction views well-being as one’s ability to attain desired goals and objects which staves of the experience of frustration [4]. In contrast, qualitative research findings have documented how individuals describe their well-being more dynamically within their everyday activities that are personally meaningful, as they adapt to changes in their health or to life stressors, and according to the degree to which they feel existentially secure in their life circumstances [5, 6]. The dynamic nature of self-reported well-being has been demonstrated by response shift across repeated assessments. Individuals are observed to adjust the internal standards by which they measure a given feature of this construct, or they reprioritize different dimensions according to adjusted personal aspirations at a given point in time, or they reconceptualise the essential definition of well-being to one that is personally relevant [7].

Evidence regarding how an individual’s appraisal of personal well-being is dynamic and shifts over time, raises an important question about how it is maintained or improved as personal aspirations evolve. Sheldon et al. have reported that individual activities are commonly informed by one’s salient aspirations [8]. These goal-directed activities are associated with positive outcomes for psychological or physical health or social well-being when they reflect intrinsic values [9]. Recent research on goal-directed activities associated with well-being was also reported in the national Midlife in the United States (MIDUS) project, where self-reported well-being was operationally defined by activities that comprised that individual’s experience of having ‘good days’ by asking the question: “What do you do to make life go well?” [10]. Response to this open-ended question was consistently associated with health behaviours and positive clinical parameters.

However, there are limited empirical data on the different types of goal-directed activities that may contribute to the pursuit of well-being. In this exploratory study, our aim was twofold: (i) to identify goal-directed activities that participants regularly engage in, which were reported to contribute to their appraisals of well-being, (ii) and to propose the prototypical categories of these reported activities to inform a novel process-based model for well-being.

## Methods

### Study design and participant selection

Eligible participants were residents of Singapore who met the following inclusion criteria: age 30–60 years, without a diagnosis of type 2 diabetes, heart disease, or kidney disease. This study was embedded within a larger qualitative study exploring the beliefs and perceptions of chronic diseases and preventative lifestyle practices among healthy adults in Singapore [11]. Hence, the inclusion criteria follows the age range of those often targeted for chronic disease prevention, excluding those who may already be diagnosed with these conditions. Following this eligibility criteria was beneficial for this study as it allowed us to identify the goal-directed activities engaged in by relatively healthy individuals. Posters and e-posters were circulated in primary care clinics and social media platforms such as Facebook and WhatsApp. Interested participants were asked to sign up and provide their age, ethnicity, education level and housing. From this pool, we used purposive sampling to ensure an ethnically diverse group of participants.

### Study procedures

Semi-structured interviews were conducted in English, Mandarin, Malay, or Tamil on a video conference call due to COVID-19 regulations. The non-English interviews were conducted by three other researchers, two of which were PhD students with qualitative methods expertise. The third researcher was extensively trained by the first author with multiple practice rounds. The overall interview duration for each participant was designed to be approximately 60 min, including questions and probes from the larger study. The section of the interview guide designed to answer this research question was about how their lives may be impacted if they were to be diagnosed with a chronic disease. To start that section, the interviewer would first encourage the participants to reflect on how they evaluate if their personal well-being was satisfactory in the context of their everyday life. As a follow-up, they would ask the participants: “What are the things or activities you have done in the last 2 weeks that made you feel like you had a good day?” inspired by the MIDUS project [10]. The interviewer would clarify that a ‘good day’ contributes to their evaluation of their personal well-being. Probes were used to understand why the activities cited were important to participants, and why or how these activities contributed to the appraisal of having “a good day”. We chose a two-week timeline for participants to recall their activities for two reasons: (i) to facilitate recall of their usual routine in case the previous week was atypical, and (ii) it was recent enough to facilitate recall with reasonable accuracy. With 40 participants, we reached saturation as no additional thematic content was gathered from our semi-structured interviews.

### Analysis

Interviews were audio-recorded, transcribed verbatim, translated for non-English interviews, and entered in NVivo 12. The non-English interviews were translated by the same researcher who conducted the interviews to ensure meaning was not lost in translation. A thematic approach was used for analysis. JH and HY worked independently to identify preliminary codes on the first 10 interviews before discussing the initial themes used to identify the types of goal-directed activities reported to contribute to a ‘good day’. A code was applied to activities that were intentionally carried out for the same outcome. For example ‘individual self-care’ was assigned to all activities described to provide time for oneself, tranquillity, and/or caring for their own physical/mental health. JH applied the initial coding scheme to the remaining interviews, which were also used to assess data saturation. The identified goal-directed activities were then categorised into themes using framework analysis [12]. RPN and JH had multiple meetings to check reflexivity of interview content and to discuss potential theoretical categories related to personal well-being. The final emergent themes were discussed by team members with expertise in quality of life research (RPN), person-centered care (TES), and qualitative research (HY and FPS).

## Results

We interviewed 40 participants, with 60% reporting a gender identity as women. The diversity in ethnicities of our sample was comparable to the Singapore population. Background characteristics of participants are outlined in Table 1. There was a larger proportion of participants with higher education than is observed in the Singapore population. Eight types of goal-directed activities that contributed to participants’ appraisal of having a “good day” emerged from the data. These activities are further elaborated below with selected quotes presented as examples.

**Table 1:** Background characteristics

Demographic Category	Count	Percentage (%)	Reference: Singapore Population (%)
**Gender**			
Female	24	60	52
Male	16	40	48
**Ethnicity**			
Chinese	29	72.5	75
Indian	4	10	10
Malay	5	12.5	12
Others	2	5	3
**Age**			
30–40 years	18	45	33
41–50 years	12	30	34
51–60 years	10	25	33
**Highest Education**			
Secondary School	6	15	40
Post-Secondary	11	27.5	26
University	23	57.5	35

**Fig. 1:**
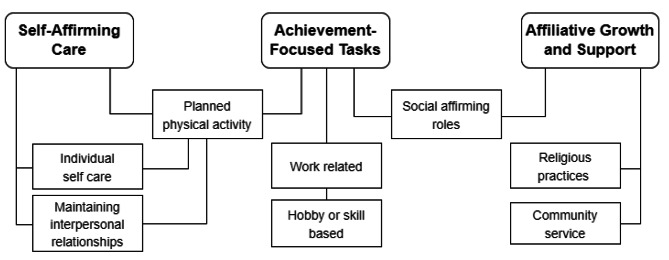
Outline of the eight goal-directed activities categorized under three primary themes contributing to living well

The eight types of goal-directed activities were categorised into three major themes (Self-Affirming Care, Achievement-Focused Tasks and Affiliative Growth and Support). Figure 1 illustrates how the eight types of activities were connected to the three major well-being themes. Self-Affirming Care comprised of types of activities that evoke self-relaxation and enjoyment independently or with others. Achievement-Focused Tasks was associated with the sense of personal accomplishment, mastery of select behaviours or fulfilment of a role. Affiliative Growth and Support referred to activities that signalled one’s contribution and connection to a social group in society at large.

It is important to note that many participants reported goal-directed activities that were characterized by multiple themes. Many of these cross-category activities were prioritized over other activities within weekly schedules. Prioritization of certain types of activities were referenced as ‘right now’ suggesting that prioritization is context-dependent and can change over time.

*“Hanging out with my girlfriend, go and eat with my friends, playing Frisbee - meet all my friends and then, sweat it out, make me feel good. Lately, work has been quite stressful, so all these extra activities is something that I always look forward to at the end of the week, or each work day. Managing my mood is something that I love, because at work, it is a very stressful environment.” (M, 30s, Malay)*.

*“Religion, family, and work are the three main things right now, and with family comes my children as well. So those three are my main that I need to fulfil then I am very happy, then other kinds of activities come next.” (F, Indian, 30s)*.

Each of the eight types of activities are further elaborated henceforth with corresponding quotes.

### Individual self care

Activities that promoted self-care, relaxation, and getting free time to do things for themselves were categorized under individual self-care. This included kid-free time for parents, time to go for a walk or jog after work, and cooking a meal for their family. These behaviours were often described as promoting a sense of tranquillity and peace, associated with stress-free time.

*“Now as a working mom, I seldom get time for myself to do what I want, like even to go to the washroom for a prolonged period of time. I just need some alone time. I feel without ‘me time’, you can’t function.” (F, 30s, Indian)*.

*“Mindfulness is one. You can do it wherever - you can lie down, or practicing yoga. Forget everything, only concentrate on breathing. Breathe in breathe out, ah, 10 seconds.” (M, 40s, Others)*.

### Maintaining interpersonal relationships

Activities directed to maintaining interpersonal relationships were centered on socializing in order to feel connected with others and to cultivate meaningful relationships. Activities ranged from meeting friends for meals to spending time with family as a channel for enjoyment and unwinding after a week of work. Time with family was mentioned with marked affection and gratefulness.

*“I think my time spent with my family, my daughter, my own mom. I think family time is important, precious.” (F, 50s, Chinese)*.

*“During the weekdays, it’s working. [During the weekends] it is something different and I will be able to talk with friends, reconnect, sometimes even connecting with new people. Then spending time with my loved ones as well” (M, 30s, Chinese)*.

### Planned physical activity

Planned physical activity was a prominent type of activity that contributed to two major themes: Self-Affirming Care and Achievement-Focused Tasks. In regard to the theme of Self-Affirming Care, physical activity exemplified self-directed effort to care for oneself that made one feel good and healthy. In regard to ‘Achievement-Focused Tasks’, physical activity promoted the experience of feeling accomplished, especially when they reported an ability to reach personal goals.

In addition to the act and benefit of exercise itself, participants mentioned the associated feelings of stress-relief and the opportunity for a shared activity with friends and family. Hence, in Fig. 1 we have shown links between physical activity and two other types of activity: individual self-care and maintaining interpersonal relationships.

*“I meet my friends to gym together and everything. I’ll consider that as a nice catch-up. I don’t really meet my friends during the weekdays, so I mean, exercising will then allow me to like, you know, hang out, chit chat, and share everything. It releases stress as well. Uh, at the same time, I think that it helps me to slim down [laughs].” (M, 30s, Chinese)*.

*“Going for a walk feels good… it just feels like I could accomplish it. When I first started walking, when [I was] not so fit, I [used to struggle when climbing] the stairs, but that day [it felt good]. And then coming back from the walk, I don’t know [if it was the] endorphins, I just feel so at ease” (F, 30s, Chinese)*.

### Work-related efficacy

Work-related efficacy was expressed when a task or initiative was successful completed, or when they managed to have a conflict-free work day. These activities were associated with a sense of progression, satisfaction, and accomplishment.

*“I teach [an instrument]. I don’t expect them to play very well but when my student progress, as long as they improve, I feel very happy” (F, 50s, Chinese)*.

*“It’s mostly career related, especially in Singapore. I think that is where we spend most of our time because the working hours are long. So then you have success in the work that you do on a day-to-day basis that is most fulfilling. When I hear some good news or when something works, that is when I would say, ‘Okay, it was a good day’.” (M, 30s, Indian)*.

### Hobby and skill-based

Efficacy to hone a skill or engage in their hobbies promoted feelings of accomplishment. Sense of satisfaction, which contributed to feelings of a “good day”, was associated with personal growth from learning or mastering a personally meaningful behaviour.

*“I’m learning how to code. [So if I can get the code right it is] pretty much quite a good day for me.” (M, 40s, Malay)*.

*“I do a craft, so recently I’m kind of obsessed with this craft, called Tsumami-zaiku. It’s a Japanese traditional craft. It doesn’t sound like fun to most people… but it takes concentration, I’m so focused on [making each piece perfect]. There is a sense of satisfaction after I’ve finished some days.” (F, 30s, Chinese)*.

### Social affirming roles

Activities fulfilling social affirming roles contributed to two major themes: Achievement-Focused Tasks and Affiliative Growth and Support. Doing house chores, cooking for the family or being able to maintain independence contributed to Achievement-Focused Tasks. These activities were reported to promote an awareness of the ability to manage social challenges or tasks, which also reinforced a sense of personal agency (i.e., putting one’s living space in ‘the right place’ within one’s life). In regard to Affiliative Growth and Support, home-based chores were reported to fulfil salient priorities related to social roles and responsibilities as a caregiver. Maintaining independence was also viewed as a means to decrease the risk of being a burden to family members.

*“I have two young kids, they have toys, loads of books. I have to sort them to donate or throw away. That makes me happy, just to clean the house. It is just to free up your mind… just simply cleaning the house and putting things in the right place.” (F, Chinese, 30s)*.

*“I want to make sure I can provide to have a good family. When you come back home, you have a family waiting for you to have dinner together. Then I can take my family on walks - with my kids.” (M, 40s, Malay)*.

*“Where you are not dependent on anybody or you are not tied down, depending on other for your daily activities. Yeah so a good day is that you can still function individually.” (M, 50s, Chinese)*.

### Religious practices

Some participants reported that attending religious activities such as church services, reading religious transcripts, or spending time with one’s respective deity contributed to a “good day”. These reported activities appeared to reinforce an existential dimension of meaning in the individual’s self-image or identity. It was common to observe reflections about finding greater purpose in life via an affiliation and connection with their deity.

*“I’m a Christian, so I’ve been doing a lot of readings and spending a lot time with God and because it’s Lent, so I’m doing the following up on the Lent seasons.” (F, 50s, Chinese)*.

### Community service

Affiliative Growth and Support was also reported through activities where an individual contributed to a charitable cause. This was expressed as giving back to society by participating in personally meaningful altruistic activities such as community services e.g., being a caretaker for orphans, or being in a role where they had the opportunity to make others feel positive.

*“We used to go to a school or a certain hospital and perform live, but now we can’t do it anymore [because of COVD-19 regulations]. So somebody is saying why don’t we just gather and perform it and record it in video? So that at least we still are able to bless people doing the same thing. Because if you’ve been performing, and then you stop doing it for six months, you’ll feel a bit empty, say, hey, I haven’t been making people smile.” (M, 50s, Chinese)*.

## Discussion

The aim of this study was to identify routine activities that were reported as comprising the appraisal of personal well-being (having a “good day”), among healthy adult participants in Singapore. Three major themes emerged from the categorization of these narrative reports: Self-Affirming Care, Achievement-Focused Tasks, and Affiliative Growth and Support.

Self-Affirming Care was reported to promote a holistic sense of well-being through activities that evoked positive mood and emotions, such as calmness, relaxation, and satisfaction or happiness within intrapersonal and interpersonal relationships. A hedonic feature of well-being is evident in these activities insofar as they were balanced towards positive (vs. negative) experiences. At the same time, a distinct feature of these activities was that they were personally meaningful because they helped to nurture or reinforce a sense of personal agency, which was expressed as taking care of oneself. This feature aligns with research on the importance of self-care in promoting well-being [13]. It was noteworthy that Self-Affirming Care activities were intentionally undertaken, which suggests that they were intrinsically meaningful to participants. This is consistent with the notion of (i) satisfying a basic psychological need for autonomy, as is well-described within self-determination theory [14], and (ii) expressing the capacity for self-determined initiatives, as is noted within self-efficacy theory [15]. Self-Affirming Care was also associated with activities where participants could feel emotionally connected to significant others to maintain interpersonal relationships. Research show that perceived social support and secured attachment with significant others are associated with therapeutic benefits, such as reduced stress, improved coping skills, and enhanced immune functioning [16, 17]. Individuals who feel a sense of connection to others also report higher levels of happiness and life satisfaction [18].

Achievement-Focused Tasks, particularly the belief in one’s ability to accomplish a task in a manner that reinforces the appraisal of ‘mastery’, is a core feature of social cognitive theory and self-determination theory [19, 20]. Activities undertaken in this category were distinct in being self-initiated, and directed towards a personally salient goal or priority. This feature reflects how Achievement-Focused Tasks are linked to personal autonomy as both a causal factor and outcome. The theme of eudaimonic well-being was a central feature of activities within this theme. Successful completion of these activities was reported to evoke a sense of personal accomplishment, acquired skill, and performance satisfaction which is consistent with flourishing that is cited as a component of well-being [21, 22]. Interestingly, participant reports also portrayed efficacy as a process. The *process* of engaging in select goal-directed activities appeared to promote personal satisfaction and self-efficacy to a degree that equalled the achievement of the intended outcome for these activities. This was particularly evident in reports of being able to maintain a smooth or conflict-free day while engaging in work activities, or being able to help a (music) student make progress in acquiring the skill of playing music, even though the students’ skill level remained below the ultimate goal of playing well. These reports are consistent with insights from clinically applied theories such as Motivational Interviewing [23], the Transtheoretical Model of readiness for change [24], and social cognitive theory [19]. Here, efficacy is described as a therapeutic process that mediates improvement in well-being or behaviour change when performance-based feedback is validating for the individual. Ryan & Deci (2017) extend this clinical insight with evidence that progress towards therapeutic outcomes is optimized when performance-based feedback is associated with change goals that are intrinsically valued by the individual (e.g. improving self-esteem or ability to be empathic), as opposed to being an extrinsic goal (becoming wealthy or socially popular) that is operantly associated with a valued outcome (e.g. happiness) [14].

Activities that were associated with Affiliative Growth and Support reflected a perceived relationship with a social organization or entity. Absence of social affiliation with community groups, referred to as social isolation, has shown strong association to higher health risk across the life course [25]. Previous research in the Singaporean population has shown that affiliation with religious organizations in the community is associated with increased mental health, including perceived emotional support and coping ability [26]. Similarly, research with vulnerable populations such as refugees indicates that perceived acceptance or support from the community at large is associated with increased psychological resilience and decreased levels of distress [27]. Activities in the Affiliative Growth and Support theme also reflected altruistic goals associated with the fulfilment of a social responsibility or role that was considered meaningful, such as a parental role, or volunteering for a cause. These activities are consistent with recent research on social “mattering”, which is the feeling of being valued by others or the perceived ability to make a valued contribution to a social group or entity. Prilleltensky et al. (2023) reviewed evidence for a reciprocal association between activities that provide valued benefit to others and the perception of social mattering, with a subsequent positive effect on perceived self-worth, as well as well-being and physical health [28].

Findings from this qualitative study provide insight into some of the distinct dimensions of goal-directed activities through which personal well-being is pursued and maintained. These findings are consistent with a process-based model that was recently introduced in the context of promoting health-related quality of life [4]. In this model, personal well-being is viewed as an appraisal that is in ongoing flux as it refers to the individual’s active process of responding to dynamic changes in and across bio-psycho-social domains of their environment. Our findings are also consistent with Alexandrova’s (2017) call to balance abstract, normative theories of well-being with mid-level theories that address how it is understood by individuals during their situation-specific life events [29]. The findings of this paper may shed some light on this research objective by focusing on salient activities that are self-reported by our participants as contributing to their personal well-being.

The study of goal-directed activities that contribute to or comprise the appraisal of personal well-being can potentially inform the development of an assessment to measure process-based approach to well-being. This can be extended to the design of interventions such as those which use behavioural activation in cognitive behavioural therapy, to promote adaptive adjustment to chronic medical conditions. There is a potential to further improve therapeutic outcomes with behavioural activation by structuring this procedure according to fundamental categories of goal-directed activities that are associated with an individual’s personal well-being, as outlined in the present study. Our recruitment site of Singapore was noteworthy because it provided an opportune cultural setting in which different Asian cultures are integrated, and from which our observations have the potential to be readily generalized. However, further exploration and quantitative assessment will be needed to confirm the reliability or to extend the scope of the present findings.

### Limitations

The findings from this exploratory study are limited in their generalizability. Firstly, the proportion of participants with higher education is much larger than in the Singapore population. This may suggest that our sample does not appropriately capture some of the experiences of lower socio-economic groups. This is important to note as a process-based approach to well-being suggests the adaptive nature of addressing adversity. Hence those who may be facing financial challenges may have a set of goal-directed activities that are not appropriately captured in this study.

Secondly, the eligibility criteria exclude key demographic groups. Our participants represent a relatively healthy population with no history of heart disease, diabetes, cancer or chronic kidney disease. Adversity in terms of health status changes can also significantly impact a process-based approach to well-being that will be important to explore in future studies. Comparing the differences in the activities reported by healthy individuals and those managing a chronic disease could provide useful insight used for clinical decisions. The study was limited to 30-60-year-old participants excluding young adults and older adults, with an over-sampling of those in their 30s. Children and young adults have very different priorities for living well and older adults living with co-morbidities may experience specific challenges in daily living which may produce a different set of goal-directed activities. Future work can benefit from looking at how goal-directed activities differ among participants in different ages or life stages.

Thirdly, since this study was conducted during the COVID-19 pandemic, the physical and social restrictions may have influenced the reported activities. While there was no mandated lockdown during the period of the interviews (late 2020-early 2021), there were restrictions for social gatherings and travel and mandated mask-wearing, which may have influenced the types of activities one engaged in. There may also have been an increase in free time, due to remote working arrangements, to participate in activities. Free time, in the context of competing responsibilities, was reported to be a big barrier to engaging in activities. Capturing this data in the post-pandemic world can provide insight into how global events like COVID-19 impact individual lives and inform future policy decisions for similar situations.

Given that these interviews were conducted over video conference calls there is a potential for social desirability bias in two instances. Firstly, even though participants were recommended to set up in a private room, they may be around family or friends and feel the need to filter their responses accordingly. Secondly, there might have been some activities participants did not feel comfortable sharing with the researcher and reported more socially accepted behaviours.

## Conclusions

This qualitative study identifies some distinct dimensions of goal-directed activities to inform a process-based model of well-being, which may be more aligned with patient-reported descriptions. Eight types of goal-directed activities were identified as promoting personal well-being, which were prototypically categorized into three core themes: Self-Affirming Care, Achievement-Focused Tasks and Affiliative Growth and Support. There is close alignment with theories and concepts, such as self-determination theory, social cognitive theory, social isolation and social mattering. These findings can inform a comprehensive process-based model of well-being which can guide the development of an assessment to complement currently used patient-reported outcome measures.

### Electronic supplementary material

Below is the link to the electronic supplementary material.

Supplementary Material 1

## Data Availability

The datasets used and/or analysed during the current study are available from the corresponding author on reasonable request.
